# Identification of a Novel Nonsense Mutation in *PLA2G6* and Prenatal Diagnosis in a Chinese Family With Infantile Neuroaxonal Dystrophy

**DOI:** 10.3389/fneur.2022.904027

**Published:** 2022-07-06

**Authors:** Yongyi Zou, Haiyan Luo, Huizhen Yuan, Kang Xie, Yan Yang, Shuhui Huang, Bicheng Yang, Yanqiu Liu

**Affiliations:** Department of Medical Genetics, Jiangxi Maternal and Child Health Hospital, Nanchang, China

**Keywords:** infantile neuroaxonal dystrophy, PLA2G6, novel variant, prenatal diagnosis, whole exome sequencing

## Abstract

**Background and Purpose:**

Infantile neuroaxonal dystrophy (INAD) is a subtype of PLA2G6-Associated Neurodegeneration (PLAN) with an age of early onset and severe clinical phenotypes of neurodegeneration. Individuals affected with INAD are characterized by rapid progressive psychomotor deterioration, neuroregression, and hypotonia followed by generalized spasticity, optic atrophy, and dementia. In this case, we aimed to identify the underlying causative genetic factors of a Chinese family with two siblings who presented with walking difficulty and inability to speak. We provided a prenatal diagnosis for the family and information for the prevention of this genetic disease.

**Methods:**

Retrospective clinical information and magnetic resonance imaging (MRI) findings of the proband were collected. Trio-whole exome sequencing (WES) including the proband and his parents was performed to explore the genetic causes, while Sanger sequencing was subsequently used to validate the variants identified by Trio-WES in the pedigree. Furthermore, prenatal molecular genetic diagnosis was carried out through amniocentesis to investigate the status of pathogenic mutations in the fetus by Sanger sequencing at an appropriate gestational age.

**Results:**

The two siblings were both clinically diagnosed with rapid regression in psychomotor development milestones. Brain MRI showed cerebellar atrophy and typical bilaterally symmetrical T2/FLAIR hyperintense signal changes in periventricular areas, indicating periventricular leukomalacia, and myelin sheath dysplasia. Trio-WES revealed two heterozygous variants in *PlA2G6* associated with clinical manifestations in the proband: a novel maternally inherited variant c.217C>T (p.Gln73^*^) and a previously reported paternally inherited recurrent pathogenic variant c.1894C>T (p.Arg632Trp). These two heterozygous mutations were also detected in the younger brother who had similar clinical features as the proband. The novel variant c.217C>T was classified as “pathogenic (PVS1 + PM2 + PP3),” while the variant c.1894C>T was “pathogenic” (PS1 + PM1 + PM2 + PM3 + PP3) based on the latest American College of Medical Genetics and Genomics (ACMG) guidelines on sequence variants. Combining the molecular evidence and clinical phenotypes, the diagnosis of INAD was established for the two affected siblings. The two variants that were identified were considered the causative mutations for INAD in this family. Prenatal diagnosis suggested compound heterozygous mutations of c.217C>T and c.1894C>T in the fetus, indicating a high risk of INAD, and the parents chose to terminate the pregnancy.

**Conclusion:**

We identified a novel pathogenic mutation that broadens the mutation spectrum of *PLA2G6* and will provide clues for the molecular diagnosis of INAD. Furthermore, our study has helped to elucidate the causative genetic factors of this Chinese family with INAD effectively and efficiently by using the emerging Trio-WES strategy and providing precise genetic counseling for this family.

## Introduction

Infantile neuroaxonal dystrophy (INAD, OMIM#256600) is a major subtype of PLA2G6-associated neurodegeneration (PLAN), characterized by severe progressive neurodegeneration caused by *PLA2G6* mutations that are inherited in an autosomal recessive mode (1). Clinical manifestations may vary from individual to individual, but most cases affected with INAD are characterized by progressive psychomotor regression, neuroregression, and hypotonia evolving into spastic tetraparesis, vision impairment, and dementia, while some may present with hearing loss (2, 3). Symptoms usually begin with the age of onset from 6 months to 3-years old and premature death mostly occurs before the age of 10, which is caused by secondary complications such as aspiration pneumonia that precedes the patient's deterioration and death (4, 5). To date, there is no effective treatment for INAD, and only supportive treatments that relieve symptoms and prevent secondary complications to prolong life are available (6).

The *PLA2G6*, as the only known responsible gene for INAD, is located on the chromosome 22 at 22q13.1 and it comprises 17 exons spanning more than 69 kb (7). *PLA2G6* encodes the cytosolic Ca^2+^-independent phospholipase A2 group VI (iPLA2-VIA), which plays an important role in maintaining the cell membrane homeostasis (8). Disease-causing variants in *PLA2G6* lead to failed repair of oxidative damage to phospholipid membranes and result in adverse changes in membrane permeability and fluidity, a mechanism that may underlie the pathology of INAD (9). The majority of patients with INAD develop cerebellar atrophy that is observed with magnetic resonance imaging (MRI) at a relatively early age (10). An initial diagnosis can be established based on the clinical manifestations and neurophysiologic, neuroradiologic, and invasive biopsy neuropathologic findings of the skin, sural nerve, or muscles (11). Over the years, with the development of DNA sequencing technologies, an increasing number of cases with INAD and pathogenic variants in *PLA2G6* have been identified effectively, allowing for efficient genetic confirmation of the diagnosis. *PLA2G6* was firstly identified as a causative gene of INAD in 2006 (1). Referring to the online database Orphanet Reports Series (https://www.orpha.net), the prevalence of the disease is unknown, but more than 150 cases have been described, most of which are classic INAD. According to the database professional Human Gene Mutation Database (HGMD), over 200 mutations in *PLA2G6* have been reported. A total of 218 variants in *PLA2G6* have been reported and more than 130 variants causing INAD have been described. Since China is a country with diverse and large populations, more investigations on the spectrum of *PLA2G6* causing INAD are needed. However, there have been only six reports on individuals affected with INAD in China so far (12–17).

Herein, we determined the genetic causative factors and a novel mutation in a Chinese family with INAD by whole exome sequencing (WES). We also describe ethical issues in this case, regarding genetic counseling for the prevention of INAD, including prenatal diagnosis.

### Case Descriptions

Two Chinese siblings who were children of a non-consanguineous healthy couple were admitted to the pediatric department in Jiangxi Maternal and Child Hospital (Jiangxi, China). They presented with delayed motor and intellectual development. There was no significant family history of neurological or heritable diseases. The two siblings were born at term after an uneventful pregnancy and delivery. No feeding difficulties or hypotonia were noticed in both of them at birth and they achieved normal developmental milestones. According to the description of their parents, the elder brother was presented with slurred speech and walking difficulty at the age of two. The proband was investigated with brain MRI, electroencephalogram (EEG), and electrocardiogram (ECG) at the age 2.5 years. The MRI revealed bilateral and symmetrical T2/FLAIR periventricular hyperintensities, consistent with periventricular leukomalacia and myelin sheath dysplasia, while ECG and EEG showed no significant abnormalities. Despite 1 year of rehabilitation, the proband had relentless symptomatic progression, with difficulty in standing balance and dysarthria. He also developed hypalgesia, emotional instability, impaired concentration, a scissoring gait, as well as developmental retardation, and regression. His muscle strength decreased to Grade 3 and showed high muscle tension, while tendon reflexes were normal. No strabismus, nystagmus, or hearing loss were observed. Follow-up MRI at the age 3.5 years old revealed an increased burden of symmetrical T2/FLAIR periventricular hyperintensities, as well as the presence of cerebellar atrophy. A repeat EEG was again unremarkable. At age 4.5 years, progressive cerebellar atrophy and new T2 hyperintensities were seen involving the bilateral frontal-parietal white matter, claval hypertrophy, with normal gray-white matter differentiation, no corpus callosum abnormalities, or evidence of brain iron accumulation. By age 6 years old, he was unable to walk or stand, with evidence of a spastic tetraparesis and axial hypotonia, and became wheelchair-bound at this time.

**Figure 1:**
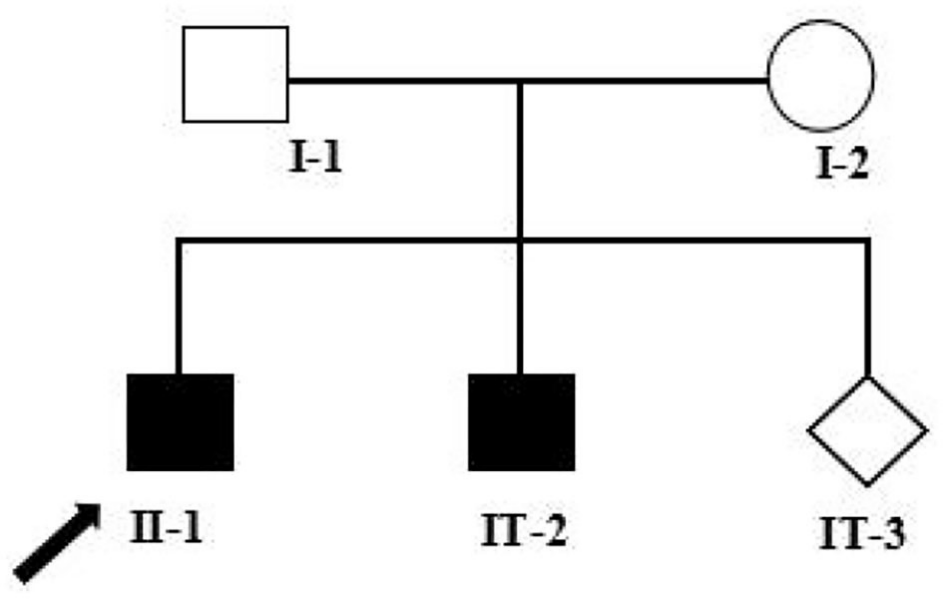
Pedigree analysis of this family. Two filled squares represent male INAD patients, unfilled square (male), and circle (female) represents healthy individuals, and rhombus represents a fetus with unknown gender. Black arrow represents the proband (II-1). I-1: Father of the proband, 39 years old; I-2: Mother of the proband, 38 years old; II-1: The proband, 7 years old; II-2: The elder brother of the proband, 5 years old; II-3: The fetus, at a gestational age of 18 + 5 weeks.

His younger brother had a similar presentation, albeit with earlier development of slurred speech at age 18 months and difficulty in walking by 4 years old, together with the gait abnormalities. The elder brother and his younger brother were separately at an age of 7 and 4 years at the time of our writing. Their mother had an ongoing pregnancy at this time and wished to have a healthy child. She, therefore, presented to the Medical Genetics Department for prenatal genetic counseling. The pedigree chart in this family is shown in Figure 1. Informed consents were obtained from the parents for the use of clinical information and samples collection involved in this study, and the study was approved by the Ethics Committee of Jiangxi Maternal and Child Health Hospital.

## Methods

### Trio-WES

Peripheral blood was extracted from the proband and both his parents after obtaining informed written consent for participation. The study was approved by the Ethics Committee of Jiangxi Maternal and Child Health Hospital. Genomic DNA was extracted from leucocytes according to the manufacturer's protocol. A NanoDrop spectrophotometer (Thermo Scientific, Wilmington, DE, USA) was used to qualify and quantify the DNA. First, WES libraries were prepared from 3 μg of genomic DNA sheared by the ultrasonication (Covaris S220 Ultrasonicator) as described previously. Then the targeted regions, including exons and splicing sites of more than 20,000 genes in the human genome, were captured and enriched with the BGI V4 chip, after which sequencing was performed with the MGISEQ-2000 platform (BGI, Shenzhen, China) with paired-end sequencing according to the manufacturer's instructions. The quality control index of sequencing data met the demand as following: the average depth of coverage for the targeted area was ≥180 ×, and the proportion of sites with an average depth >20 × of the targeted area was >95%. The sequenced fragments were aligned to the UCSC RefSeq database hg19 human reference genome by BWA to remove duplications. A unified genotyper tool from GATK was then used for variant calling of single nucleotide variations, insertions, or deletions (Indels) and copy number variations at the level of exons by the Exome Depth based on probability-based and quality-based algorithms. Briefly, common polymorphisms in the dbSNP (minor allele frequency > 0.01), 1,000 genomes (genotype frequency > 0.005), EVC, and gnomAD, as well as synonymous single nucleotide variants were filtered out.

### Variant Annotations and Interpretations

The gene nomenclature follows the conventions of the Human Genome Organization Gene Nomenclature Committee; the variant nomenclature follows the conventions of the Human Genome Variation Society. Variant annotation and screening were based on clinical phenotypes of the subject, population database (dbSNP, 1,000 Genome, ExAC), disease database (OMIM, HGMD, Clinvar), and biological information prediction tools (SIFT, Polyphen2, and Mutation Taster). The interpretation of pathogenicity for variants was based on the guidelines by the American College of Medical Genetics and Genomics (ACMG) and the American Society for Molecular Pathology. Detailed interpretations of the guidelines were referred to the ClinGen Sequence Variation Interpretation Working Group and the British Society of Clinical Genomics (ACGS). When a rare variant predicted to be deleterious was observed in a single known cerebellar atrophy gene and associated with a correspondent mode of inheritance, it was considered as a potential candidate.

### Confirmation of the Two Variants by Sanger Sequencing

To verify the whole exome sequence results and segregation analysis, Sanger sequencing was performed to identify the candidate variants for the two patients and their parents using the 3500DX Genetic Analyzer (Applied Biosystems). The Seqman software (Technelysium, South Brisbane, QLD, Australia) was used for the sequence analysis with standard reference sequences and visualizations. The reference sequences were obtained using the online database at the University of California, Santa Cruz (UCSC). Two distinct primer pairs were designed and primer sequences for validating the two detected mutations are listed in Table 1.

**Table 1:** Two primers used for the Sanger sequencing confirmation.

**Primer names**	**Sequences (5^′^ → 3^′^)**	**Product length (bp)**
PLA2G6-E3F	CCCGCCTTACTCATTTCGGG	356
PLA2G6-E3R	CAAACTATGGAGGGGAACCGAG	
PLA2G6-E14F	ACCAGGACGAACTAGCCAGA	597
PLA2G6-E14R	GTGGCACGTTCATGGTATGC	

### Prenatal Diagnosis of the Fetus

After the causative mutations were identified for the two siblings, the parents expressed a desire to undergo prenatal diagnosis for the fetus at an appropriate gestational week. A total of 20 ml of amniotic fluid samples were collected from the pregnant mother under the guidance of B ultrasonography at the gestational age of 18W + 4. The extraction of fetal DNA from the amniotic fluid was conducted using a QIAamp DNA Mini Kit (Qiagen, Germany), according to the protocols of the manufacturer. Forward and reverse sequencing was applied to investigate the mutation status of c.217C>T and c.1894C>T in *PLA2G6* and the sequencing results were analyzed as described previously. Meanwhile, the elimination of maternal cell contamination was carried out by the quantitative fluorescent polymerase chain reaction, according to the protocol described previously (18).

## Results

### Whole Exome Sequencing Findings and Sanger Sequencing Validation

The sequencing production of raw data by WES reached at least 23,170.74 Mb for each candidate. Approximately 99.72% of the sequencing reads were mapped to the human genome hg19, with a mean 273.8.5 × sequencing depth. Interestingly, two compound heterozygous disease-associated variants in *PLA2G6* (NM_003560) in the proband, a maternally inherited variant c.217C>T and the paternally inherited variant c.1894C>T, passed the filtering criteria. For these two variants, variant c.217C>T in exon 3 results in a premature stop codon with early termination of protein translation at residue 73 (p.G73X), while variant c.1894C>T in exon 14 leads to a change of amino acid Arg by Trp (p.Arg632Trp). Subsequently, Sanger sequencing validated that the two mutations co-segregated with the disease in the family. These two heterozygous mutations were also detected in the younger brother of the proband affected with INAD.

### Variant Interpretations and Interpretations According to the ACMG Guidelines

**Figure 2:**
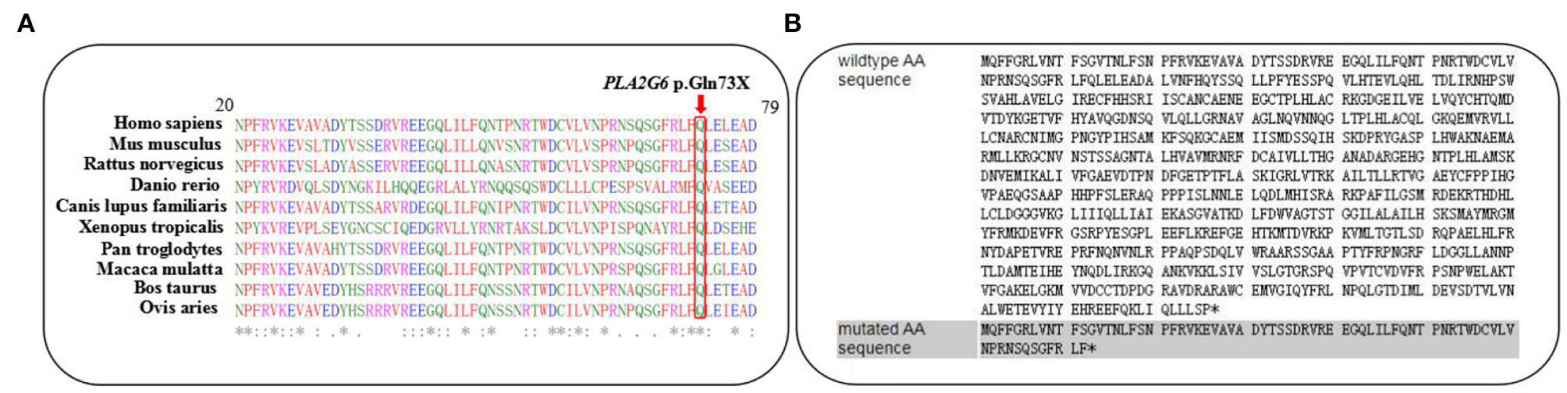
**(A)** The changed amino acid Q73, indicated by a red arrow, is highly evolutionarily conserved among species. **(B)** Predicted premature termination codon at the 73rd residue of iPLA2-VIAβ encoded by *PLA2G6*.

The variant c.217C>T was not observed in the 1,000 Genomes database, the Exome Variant Server, Genome Aggregation Database (gnomAD), or in the HGMD professional database. On the other hand, it was not detected in our 200 healthy control cohorts using high-resolution melting analysis (SsoFast EvaGreen, Bio-Rad). It is a nonsense variant predicted to lead to a substitution of glutamine with an immediate stop codon introduction at the 73rd residue of IPLVII-β (p.G73^*^). Alignments of the PLA2G6 protein family members by the ClustalX online software revealed that the 73rd amino acid glutamine of iPLA2-VIA is highly evolutionarily conserved, as shown in Figure 2A. We speculated that this novel mutation produces altered mRNA harboring, a premature termination codon that will be selectively degraded by the nonsense-mediated mRNA decay. This novel variant is predicted to be deleterious by multiple bioinformatic algorithms, such as SIFT, polyphen-2, and Mutation Taster. According to the latest ACMG guideline, the novel variant c.217C>T is classified as pathogenic (PVS1 + PM2 + PP3). In our present study, the detected missense variant c.1894C>T leads to a change of amino acid Arg by Trp. According to the ACMG guidelines, this variant is classified as pathogenic (PS1 + PM1 + PM2 + PM3 + PP3). Combined with the clinical features, genetic analysis confirmed the diagnosis of INAD in this family.

### Genetic Findings for the Fetus by Amniocentesis

**Figure 3:**
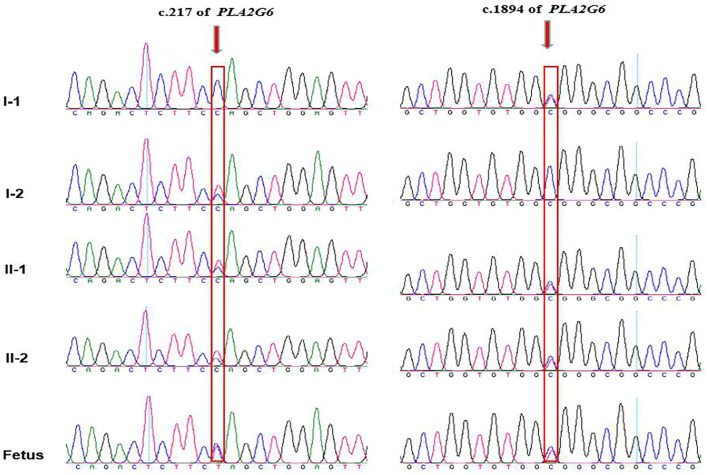
Chromatograms of the two mutations detected in the family by Sanger sequencing. The proband and his elder brother are compound heterozygous with inherited maternal c.217C>T and inherited paternal c.1894C>T, while the fetus are detected with the same compound heterozygous mutations as the proband. Arrows indicates mutation site.

Amniotic fluid testing after an additional 7 days revealed that the fetus was compound heterozygous with both mutations, harboring the same genotype as the proband. The chromatograms of Sanger sequence analysis for the family members are shown in Figure 3. After adequate consideration, the family decided to terminate the pregnancy at the gestational age of 20 + 4 weeks. All the chromatograms of Sanger sequence analysis for the family members are shown in Figure 3. After adequate consideration, the family decided to interrupt the pregnancy as early as possible.

## Discussion

**Figure 4:**
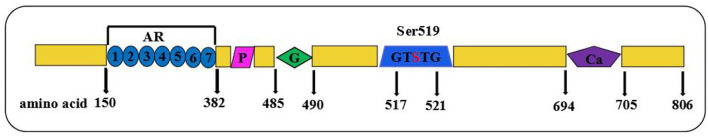
Schematic showing full-length of protein structure of iPLA2-VIA. Domain composition of iPLA2-VIA. AR domain indicating even ankyrin repeats are shown in blue circles; P indicates a proline-rich motif (purple rhomboid) domain; G domains indicates the poly-Gly region is in green diamond, S indicates the lipase motif Ser519, and Ca indicates putative CaM-binding motifs in purple pentagon.

*PLA2G6* encodes iPLA2-VIA, an 85/88 kDa calcium-independent phospholipase belonging to the phospholipase A2 family, which catalyzes the hydrolysis of the sn-2 fatty acyl bonds in phospholipids, generating lysophospholipids, and free fatty acids. As the longest one among the five transcripts, iPLA2-VIA-2 contains 806 amino acids and plays an important role in regulating important physiological processes, such as inflammation, calcium homeostasis, and apoptosis in various cell types (19). The full length of iPLA2-VIA consists of seven N-terminal ankyrin-like repeats, a proline-rich motif, a glysine-rich nucleotide-binding motif, a lipase motif, and a binding site for calmodulin at the C-terminus region (19), as shown in Figure 4. A seven ankyrin-like repeats domain is involved in enzyme oligomerization, promoting the expression of full enzymatic activity, while three other domains are involved in the regulation of enzymatic activity and lipase motif responsible for the protein phosphorylation (7, 20–22). According to previous studies, mutation sites in the ankyrin repeat domains, catalytic (CAT) domains, or any other domains may lead to different enzyme activities (23). In our case, a premature stop codon at the 73rd amino acid directly causes an end of the essential structures of iPLA2-VIA proteins. A complex group of PLAN encompasses infantile neuroaxonal dystrophy (INAD), atypical neuroaxonal dystrophy (ANAD), parkinsonian syndrome containing both adult-onset dystonia parkinsonism (DP), and early onset parkinsonism (AREP) based on the age of onset and progressive clinical manifestations (24, 25). INAD differs from three others with the characterization of the earlier age of onset within 3 years and distinctive clinical features (26). Most notably, INAD and ANAD are two subtypes of PLAN that may share some similar clinical phenotypes, brain MRI/image signs, and treatment strategies, while ANAD is usually characterized by the onset outside the infantile period in early childhood and the more heterogeneous phenotypes (26). Compared with INAD cases, the age of onset for ANAD ranges from 3 years old to the late teens and ANAD patients mostly present with a slower progression and longer survival times (27).

The majority of these INAD cases were detected with direct sequencing for coding regions of *PLA2G6*, which is time-consuming and inefficient. In reported studies involving the Chinese population, 26 variants have been identified, as listed in Table 2. Most of these variants were missense mutations, while the remaining were frame shift or nonsense mutations, which lead to loss of protein function. Our case provides a further nonsense mutation. However, further investigation on the underlying genetic mechanisms of INAD caused by PLA2G6 mutations is needed.

**Table 2:** *PLA2G6* mutations previously reported in the Chinese INAD patients and the relevant literature.

**Numbers**	**Nucleotide change**	**Exons**	**Amino acid change**	**Variation type**	**Related PMID**
1	c.171C>A	Exon2	p.C57*	Nonsense	22934738
2	c.208C>T	Exon2	p.R70*		19138334
3	c.858C>G	Exon6	p.Y286*		22934738
4	c.2266C>T	Exon16	p.Q756*		31922589
5	c.2389C>T	Exon17	p.Q797*		22934738
6	c.27_28insA	Exon2	p.T10Nfs*9	Frameshift	22934738
7	c.28dupA	Exon2	p.T10Nfs*11		22934738
8	c.373delC	Exon3	p.L125Wfs*5		22934738
9	c.496dupG	Exon4	p.E166Gfs*32		30112060
10	c.2060delT	Exon15	p.L687Pfs*17		22934738
11	c.1A>G	Exon2	p.M1V	Missense	19138334
12	c.68G>A	Exon2	p.R 23Q		26829737
13	c.116G>A	Exon2	p.R39Q		19138334
14	c.692G>T	Exon5	p.G231V		22934738
15	c.1111G>A	Exon8	p.V371M		19138334
16	c.1117G>A	Exon8	p.G373R		19138334
17	c.1496C>T	Exon11	p.A499V		22934738
18	c.1610T>A	Exon12	p.M537K		22934738
19	c.1633A>G	Exon12	p.K545E		19138334
20	c.1771C>T	Exon13	p.R591W		19138334
21	c.1957G>A	Exon14	p.G653S		22934738
22	c.1970C>T	Exon14	p.A657V		19138334
23	c.1984C>G	Exon14	p.L662V		31506141
24	c.2261G>T	Exon16	p.G754V		22934738
25	c.1427+1G >A	Intron 10	/	Splice	19138334
26	c.1743-1G>T	Intron 11	/		31506141

*Indicates the stop of peptide synthesis.

Infantile neuroaxonal dystrophy was firstly described by Seitelberger in 1952 and INAD patients often show slight psychomotor and dystonia disorders during infancy and childhood (28, 29). Other clinical manifestations may include bilateral limb spasticity, bulbar signs (impaired swallowing and dyspnea), pendular nystagmus, strabismus, distal contractures, optic atrophy, and hearing impairment (30, 31). These symptoms usually result in total neurological degeneration and mortality in the first decade of INAD suffers (32). The majority of patients with INAD develop cerebellar atrophy appreciable on MRI at a relatively early age. In addition, INAD cases exhibit spheroid bodies in the central nervous system and pathological swelling of axons in their neuropathology. Therefore, MRI images and the presence of high brain iron or optic atrophy are considered to be suggestive findings for the diagnosis (4, 5). According to Nardocci et al., a series of clinical, neurophysiologic, neuroradiologic, and invasive biopsy neuropathologic findings are essential to establish a diagnosis of INAD (33). Our cases presented with the typical clinical features. In a large case series of 28 INAD patients, speech impairment and loss of gross motor milestones were the most common signs of the disease, while there is phenotypic variability, with other common clinical findings such as nystagmus (60.7%), seizures (42.9%), gastrointestinal disease (42.9%), skeletal deformities (35.7%), and strabismus (28.6%) (2). In a further study of 24 Chinese INAD patients, nystagmus was observed in eight and strabismus in two patients (12). The combination of typical clinical features and genetic testing in the present report were consistent with a diagnosis of INAD. However, it is notable that the proband did not have evidence of strabismus, nystagmus, or generalized fast rhythms in EGG, as has been described (6). Therefore, we assumed that the case in our report also showed heterogeneous phenotypes. In 2006, *PLA2G6* was firstly identified as a causative gene for INAD by Morgan, after which molecular testing was promoted as the definitive diagnosis of INAD and this eliminated the need for invasive biopsies, while also enabling the detection of carriers of INAD-associated mutations and allowing for prenatal diagnosis and pre-implantation genetic diagnosis (19, 34). Despite sharing only ~2% of the human genome, the exome harbors nearly 85% of mutations with large effects on disease-related traits (35). In recent years, WES has quickly emerged as the most widely used targeted enrichment method, particularly for monogenic (“Mendelian”) diseases. In this case, WES allowed for the unbiased analysis of all genes and eliminated the need for a time-consuming selection of candidate genes prior to sequencing (36, 37).

In our study, the combination of WES and clinical history facilitated the diagnosis of INAD and facilitated effective genetic counseling and prenatal diagnosis. Since there existed a relatively high risk with a 1/4 probability of giving birth to an affected INAD child at each pregnancy for this couple, prenatal genetic diagnosis is recommended according to the Chinese legislation related to prenatal diagnosis. Furthermore, induced abortion for medical indications such as the birth of a newborn with severe deformities is permitted by the Ethics Committee and Chinese legislation regulating abortion. The fetus in this family was detected with compound heterozygous pathogenic variants of *PLA2G6* and was very likely to be affected with INAD after birth, considering the relevant family clinical history. After adequate informed consent, the parents were permitted to terminate the pregnancy in line with medical indications after discussion by the hospital ethics committee at a gestational age of <24 weeks.

## Conclusions

We presented a novel likely pathogenic variant causing a compound heterozygous presentation of PLA2G6-associated INAD and demonstrated the clinical heterogeneity of this population. We describe how providing accurate genetic counseling in the light of pathogenic findings in the fetus led to a decision to terminate the pregnancy.

## Data Availability Statement

The original contributions presented in the study are publicly available. This data can be found in Figshare, https://figshare.com/, doi: 10.6084/m9.figshare.20188400.v1.

## Ethics Statement

The studies involving human participants were reviewed and approved by Ethics Committee of Jiangxi Maternal and Child Health Hospital (No. 20200086). Written informed consent to participate in this study was provided by the participants' legal guardian/next of kin.

## Author Contributions

YZ and YL were in charge of the genetic analysis, conceptualized the study, and wrote the manuscript original draft preparation. HY and SH were responsible for the diagnosis and clinical evaluation. BY, YY, and KX did the investigation. SH, BY, and YL wrote, reviewed, and edited the manuscript. All authors have read and agreed to the published version of the manuscript. All authors contributed to the article and approved the submitted version.

## Funding

This study was supported by the National Natural Science Foundation of China (No. 82160318) and Jiangxi Provincial Key Laboratory of Birth Defect for Prevention and Control (No. 20202BCD42017).

## Conflict of Interest

The authors declare that the research was conducted in the absence of any commercial or financial relationships that could be construed as a potential conflict of interest.

## Publisher's Note

All claims expressed in this article are solely those of the authors and do not necessarily represent those of their affiliated organizations, or those of the publisher, the editors and the reviewers. Any product that may be evaluated in this article, or claim that may be made by its manufacturer, is not guaranteed or endorsed by the publisher.

## References

[1] Khateeb S Flusser H Ofir R Shelef I Narkis G Vardi G. PLA2G6 mutation underlies infantile neuroaxonal dystrophy. Am J Hum Genet. (2006) 79:942–8. 10.1086/508572 17033970 PMC1698558

[2] Altuame FD Foskett G Atwal PS Endemann S Midei M Milner P. The natural history of infantile neuroaxonal dystrophy. Orphanet J Rare Dis. (2020) 15:109. 10.1186/s13023-020-01355-2 32357911 PMC7193406

[3] Gregory A Kurian MA Maher ER Hogarth P Hayflick SJ. PLA2G6-Associated Neurodegeneration[M]. GeneReviews [Internet]. Seattle, WA: University of Washington (1993). 20301718

[4] Gregory A Westaway SK Holm IE Kotzbauer PT Hogarth P Sonek S. Neurodegeneration associated with genetic defects in phospholipase A(2). Neurology. (2008) 71:1402–9. 10.1212/01.wnl.0000327094.67726.28 18799783 PMC2676964

[5] Kurian MA Morgan NV MacPherson L Foster K Peake D Gupta R. Phenotypic spectrum of neurodegeneration associated with mutations in the PLA2G6 gene (PLAN). Neurology. (2008) 70:1623–9. 10.1212/01.wnl.0000310986.48286.8e 18443314

[6] Guo YP Tang BS Guo JF. PLA2G6-associated neurodegeneration (PLAN): review of clinical phenotypes and genotypes. Front Neurol. (2018) 9:1100. 10.3389/fneur.2018.01100 30619057 PMC6305538

[7] Larsson PK Claesson HE Kennedy BP. Multiple splice variants of the human calcium-independent phospholipase A2 and their effect on enzyme activity. J Biol Chem. (1998) 273:207–14. 10.1074/jbc.273.1.207 9417066

[8] Tanaka H Minakami R Kanaya H Sumimoto H. Catalytic residues of group VIB calcium-independent phospholipase A2 (iPLA2gamma). Biochem Biophys Res Commun. (2004) 320:1284–90. 10.1016/j.bbrc.2004.05.225 15249229

[9] Shinzawa K Sumi H Ikawa M Matsuoka Y Okabe M Sakoda S. Neuroaxonal dystrophy caused by group VIA phospholipase A2 deficiency in mice: a model of human neurodegenerative disease. J Neurosci. (2008) 28:2212–20. 10.1523/JNEUROSCI.4354-07.2008 18305254 PMC6671850

[10] Tanabe Y Iai M Ishii M Tamai K Maemoto T Ooe K. The use of magnetic resonance imaging in diagnosing infantile neuroaxonal dystrophy. Neurology. (1993) 43:110–3. 10.1212/wnl.43.1_part_1.110 8423872

[11] Carrilho I Santos M Guimarães A Teixeira J Chorão R Martins M. Infantile neuroaxonal dystrophy: what's most important for the diagnosis. Eur J Paediatr Neurol. (2008) 12:491–500. 10.1016/j.ejpn.2008.01.005 18359254

[12] Zhang P Gao Z Jiang Y Wang J Zhang F Wang S. Follow-up study of 25 Chinese children with PLA2G6-associated neurodegeneration. Eur J Neurol. (2013) 20:322–30. 10.1111/j.1468-1331.2012.03856.x 22934738

[13] Wu Y Jiang Y Gao Z Wang J Yuan Y Xiong H. Clinical study and PLA2G6 mutation screening analysis in Chinese patients with infantile neuroaxonal dystrophy. Eur J Neurol. (2009) 16:240–5. 10.1111/j.1468-1331.2008.02397.x 19138334

[14] Wang B Wu D Tang J. Infantile neuroaxonal dystrophy caused by PLA2G6 gene mutation in a Chinese patient: a case report. Exp Ther Med. (2018) 16:1290–4. 10.3892/etm.2018.6347 30112060 PMC6090475

[15] Wang J Wu W Chen X Zhang L Wang X Dong G. A novel homozygous mutation in PLA2G6 gene causes infantile neuroaxonal dystrophy in a case. Zhonghua Yi Xue Yi Chuan Xue Za Zhi. (2016) 33:64–7. 10.3760/cma.j.issn.1003-9406.2016.01.016 26829737

[16] Lu Y Liu CH Wang Y. Clinical features of infantile neuroaxonal dystrophy and PLA2G6 gene testing. Zhongguo Dang Dai Er Ke Za Zhi. (2019) 21:851–5. 10.7499/j.issn.1008-8830.2019.09.002 31506141 PMC7390253

[17] Tan J Yan T Chang R Yuan D Pan L Cai R. Analysis of PLA2G6 gene variant in a family affected with infantile neuroaxonal dystrophy. Zhonghua Yi Xue Yi Chuan Xue Za Zhi. (2020) 37:21–4. 10.3760/cma.j.issn.1003-9406.2020.01.006 31922589

[18] Noveski P Terzic M Vujovic M Kuzmanovska M Sukarova Stefanovska E Plaseska-Karanfilska D. Multilevel regression modeling for aneuploidy classification and physical separation of maternal cell contamination facilitates the QF-PCR based analysis of common fetal aneuploidies. PLoS ONE. (2019) 14:e0221227. 10.1371/journal.pone.0221227 31430300 PMC6701765

[19] Malley KR Koroleva O Miller I Sanishvili R Jenkins CM Gross RW. The structure of iPLA(2)β reveals dimeric active sites and suggests mechanisms of regulation and localization. Nat Commun. (2018) 9:765. 10.1038/s41467-018-03193-0 29472584 PMC5823874

[20] Morgan NV Westaway SK Morton JE Gregory A Gissen P Sonek S. PLA2G6, encoding a phospholipase A2, is mutated in neurodegenerative disorders with high brain iron. Nat Genet. (2006) 38:752–4. 10.1038/ng1826 16783378 PMC2117328

[21] Mosavi LK Cammett TJ Desrosiers DC Peng ZY. The ankyrin repeat as molecular architecture for protein recognition. Protein Sci. (2004) 13:1435–48. 10.1110/ps.03554604 15152081 PMC2279977

[22] Tang J Kriz RW Wolfman N Shaffer M Seehra J Jones SS. A novel cytosolic calcium-independent phospholipase A2 contains eight ankyrin motifs. J Biol Chem. (1997) 272:8567–75. 10.1074/jbc.272.13.8567 9079687

[23] Jenkins CM Wolf MJ Mancuso DJ Gross RW. Identification of the calmodulin-binding domain of recombinant calcium-independent phospholipase A2beta. Implications for structure and function. J Biol Chem. (2001) 276:7129–35. 10.1074/jbc.M010439200 11118454

[24] Darling A Aguilera-Albesa S Tello CA Serrano M Tomás M Camino-León R. PLA2G6-associated neurodegeneration: new insights into brain abnormalities and disease progression. Parkinsonism Relat Disord. (2019) 61:179–86. 10.1016/j.parkreldis.2018.10.013 30340910

[25] Salih MA Mundwiller E Khan AO AlDrees A Elmalik SA Hassan HH. New findings in a global approach to dissect the whole phenotype of PLA2G6 gene mutations. PLoS ONE. (2013) 8:e76831. 10.1371/journal.pone.0076831 24130795 PMC3792983

[26] Illingworth MA Meyer E Chong WK Manzur AY Carr LJ Younis R. PLA2G6-associated neurodegeneration (PLAN): further expansion of the clinical, radiological and mutation spectrum associated with infantile and atypical childhood-onset disease. Mol Genet Metab. (2014) 112:183–9. 10.1016/j.ymgme.2014.03.008 24745848 PMC4048546

[27] Sadeh M. Neurodegeneration associated with genetic defects in phospholipase A2. Neurology. (2009) 73:819. 10.1212/WNL.0b013e3181b2851b 19738181

[28] Seitelberger F. Neuropathological conditions related to neuroaxonal dystrophy. Acta Neuropathol. (1971) 5(Suppl. 5):17–29. 10.1007/978-3-642-47449-1_3 5562689

[29] Gregory A Polster BJ Hayflick SJ. Clinical and genetic delineation of neurodegeneration with brain iron accumulation. J Med Genet. (2009) 46:73–80. 10.1136/jmg.2008.061929 18981035 PMC2675558

[30] Iannello G Graziano C Cenacchi G Cordelli DM Zuntini R Papa V. A new PLA2G6 mutation in a family with infantile neuroaxonal dystrophy. J Neurol Sci. (2017) 381:209–12. 10.1016/j.jns.2017.08.3260 28991683

[31] Frattini D Nardocci N Pascarella R Panteghini C Garavaglia B Fusco C. Downbeat nystagmus as the presenting symptom of infantile neuroaxonal dystrophy: a case report. Brain Dev. (2015) 37:270–2. 10.1016/j.braindev.2014.04.010 24800972

[32] Riku Y Ikeuchi T Yoshino H Mimuro M Mano K Goto Y. Extensive aggregation of α-synuclein and tau in juvenile-onset neuroaxonal dystrophy: an autopsied individual with a novel mutation in the PLA2G6 gene-splicing site. Acta Neuropathol Commun. (2013) 1:12. 10.1186/2051-5960-1-12 24252552 PMC3893443

[33] Nardocci N Zorzi G Farina L Binelli S Scaioli W Ciano C. Infantile neuroaxonal dystrophy: clinical spectrum and diagnostic criteria. Neurology. (1999) 52:1472–8. 10.1212/wnl.52.7.1472 10227637

[34] Goyal M Bijarnia-Mahay S Kingsmore S Farrow E Saunders C Saxena R. Molecular diagnosis of infantile neuro axonal dystrophy by next generation sequencing. Indian J Pediatr. (2015) 82:474–7. 10.1007/s12098-014-1608-z 25348461 PMC4390426

[35] Nuytemans K Vance JM. Whole exome sequencing. Rinsho Shinkeigaku. (2010) 50:952–5. 10.5692/clinicalneurol.50.952 21921524

[36] Singleton AB. Exome sequencing: a transformative technology. Lancet Neurol. (2011) 10:942–6. 10.1016/S1474-4422(11)70196-X 21939903 PMC3302356

[37] Petrovski S Aggarwal V Giordano JL Stosic M Wou K Bier L. Whole-exome sequencing in the evaluation of fetal structural anomalies: a prospective cohort study. Lancet. (2019) 393:758–67. 10.1016/S0140-6736(18)32042-7 30712878

