# Supplementary material for: Identification of a Novel Nonsense Mutation in PLA2G6 and Prenatal Diagnosis in a Chinese Family With Infantile Neuroaxonal Dystrophy
Source: Front Neurol. 2022 Jul 6;13:904027. doi: 10.3389/fneur.2022.904027 (PMC9298276; doi:10.3389/fneur.2022.904027)
Supplement: Supplementary file 1 [file Table_1.pdf]

Table S1. Links for the Population, Disease-Specific, and Sequence Databases

Population, Disease-Specific, and Sequence Databases	Links
UCSC	https://genome.ucsc.edu/
BWA	https://bio-bwa.sourceforge.net
GATK	https://www.broadinstitute.org/gatk/
dbSNP	https://www.ncbi.nlm.nih.gov/snp/
Exome Variant Server(EVS)	http://evs.gs.washington.edu/EVS/
Polyphen-2	http://genetics.bwh.harvard.edu/pph2/index. html
SIFT	https://sift.bii.a-star.edu.sg/
MutationTaster	http://www.mutationtaster.org/
Genome Aggregation Database (gnomAD)	http://gnomad.broadinstitute.org/
OMIM	http://www.omim.org
HGMD	http://www.hgmd.org
Clinvar	https://www.ncbi.nlm.nih.gov/clinvar
Clustal X	https://www.ebi.ac.uk/Tools/msa/clustalo/
